# Cost-Effectiveness of a Home Based Intervention for Secondary Prevention of Readmission with Chronic Heart Disease

**DOI:** 10.1371/journal.pone.0144545

**Published:** 2015-12-10

**Authors:** Joshua Byrnes, Melinda Carrington, Yih-Kai Chan, Christine Pollicino, Natalie Dubrowin, Simon Stewart, Paul A. Scuffham

**Affiliations:** 1 Centre for Applied Health Economics, Griffith University, Meadowbrook, Australia; 2 Mary MacKillop Institute for Health Research, Australian Catholic University, Melbourne, Australia; 3 Bupa Australia, Melbourne, Australia; Deakin University, AUSTRALIA

## Abstract

The aim of this study is to consider the cost-effectiveness of a nurse-led, home-based intervention (HBI) in cardiac patients with private health insurance compared to usual post-discharge care. A within trial analysis of the Young @ Heart multicentre, randomized controlled trial along with a micro-simulation decision analytical model was conducted to estimate the incremental costs and quality adjusted life years associated with the home based intervention compared to usual care. For the micro-simulation model, future costs, from the perspective of the funder, and effects are estimated over a twenty-year time horizon. An Incremental Cost-Effectiveness Ratio, along with Incremental Net Monetary Benefit, is evaluated using a willingness to pay threshold of $50,000 per quality adjusted life year. Sub-group analyses are conducted for men and women across three age groups separately. Costs and benefits that arise in the future are discounted at five percent per annum. Overall, home based intervention for secondary prevention in patients with chronic heart disease identified in the Australian private health care sector is not cost-effective. The estimated within trial incremental net monetary benefit is -$3,116 [95%CI: -11,145, $4,914]; indicating that the costs outweigh the benefits. However, for males and in particular males aged 75 years and above, home based intervention indicated a potential to reduce health care costs when compared to usual care (within trial: -$10,416 [95%CI: -$26,745, $5,913]; modelled analysis: -$1,980 [95%CI: -$22,843, $14,863]). This work provides a crucial impetus for future research to understand for whom disease management programs are likely to benefit most.

## Introduction

Management of cardiovascular disease (CVD) in Australia costs $5.94 billion annually, approximately 11% of total health care expenditure[1]. Significant financial pressure is emerging with an increasing number of older individuals surviving an acute cardiac event but with residual risk for subsequent (and often costly) events. There are significant gaps in the overall application of gold-standard secondary prevention[2] despite expert recommendations to prevent disease progression[3, 4]. Further, individual factors, such as treatment non-adherence, poor knowledge, poor health literacy and sub-optimal self-care behaviors[5] are likely to contribute to poorer longer-term impacts of traditional (and predominantly short-term) cardiac rehabilitation programs [6] notwithstanding practical issues such as insufficient access to prevention programs following an acute cardiac event[7].

The Australian health care system is a hybrid of both a public system (no patient out of pocket costs and with universal access to hospital treatment—all be it rationed through the use of waiting lists) as well as a private health industry supported by private health insurance. The private health insurance sector supports the public system through payments made on behalf of insured patients. In 2010, this was more than six hundred million dollars. Individuals with private health insurance (47% of the Australian population[8]) use more healthcare resources with a greater frequency (e.g. primary care consultations and elective hospital procedures)[9]. Within this population, the Young@Heart Study aimed to examine the benefits of a tailored disease management program.

The hypothesis of the Young @ Heart study is that a specialist nurse-led, multidisciplinary, home-based intervention (HBI) program for individuals diagnosed with a chronic form of heart disease and aged over 45 could reduce the rate of all-cause hospital days stay or all-cause hospitalization. Whilst analysis of clinical outcomes from the trial have been previously assessed[2], the aim of this study is to assess the cost-effectiveness of the Young @ Heart program for the trial period as well as provide an assessment of the programs cost-effectiveness beyond the duration of the trial based on modelled projections. Whilst overall group differences from the clinical trial were not statistically significant, the outcomes of the intervention were predominantly worse for women. Alternatively, for men, HBI was associated with reduced risk of cardiovascular-related hospitalization suggesting a sex-specific response to the study intervention. It is unknown whether the intervention represents good value for money either overall or when targeted specifically to those sub-groups for whom there was a greater treatment effect. Consequently, this analysis will explore the cost and effectiveness for sub-groups delineated by age and sex. As this intervention is targeted for patients with a chronic disease, it is important that a modelled analysis be conducted to consider the long-term cost and consequences of the intervention.

## Methods

### Young@Heart trial

The Young @ Heart Study was registered with the Australian New Zealand Clinical Trials Registry (No. 12608000014358) and conforms to both the principles outlined in the Declaration of Helsinki [10] and the CONSORT guidelines[11]. The study was approved by the human Research Ethics Committee of Uniting Care Health, Brisbane, Australia and all patients provided written informed consent to participate. The Young@Heart trial protocol and description of interventions have been previously described in full [12]. In short, the Young@Heart Study was a multi-centre, randomised controlled trial of a nurse-led, home-based intervention (HBI) program compared to usual care (UC). The HBI was offered to eligible members of a large private health insurer who were recently hospitalised for chronic heart disease. Patients (> = 45 years of age) were identified from their bedside in two hospitals. The intervention was delivered through cardiac nurses specifically employed to deliver the intervention and was tailored to an individual’s clinical stability, management and risk profile[13]. Based on their home assessments and broader clinical profile of the patient, the cardiac nurses developed a detailed report and tailored health care plan for various members of the patient's health care team as well as initiated a broad range of interventions to supplement the intervention. These included referrals to primary care physicians, community pharmacists, dieticians, diabetes educators, cardiac rehabilitation programs or other allied health care service. HBI patients were able to contact the cardiac nurse for continued advice and support and if readmitted to hospital, a repeat home visit at 7 to 14 days was implemented, accompanied by the preparation of a revised health care plan. Overall, 602 (mean age 70 ± 10 years, 72% men) patients were randomized to usual post-discharge care (UC, n = 296) or the same supplemented by HBI (n = 306–96% received at least one home visit) between 1 July 2008 and 31 December 2009.

Previously published results from the Young@Heart trial at 2 years [2], reported that 42 patients (7.0%) had died and 492 patients (82%) were hospitalized with 2,338 all-cause admissions and 10,045 days of hospitalization. There were no group differences (HBI vs. UC) in the primary endpoints of all-cause hospital days stay or all-cause hospitalization. HBI outcomes were predominantly worse for women. Alternatively, in men HBI was associated with reduced risk of cardiovascular-related hospitalization suggesting a sex-specific response to the study intervention. Consequently, analyses have been conducted on the basis of the entire trial population as well as for sub-groups delineated by age and sex.

### Within trial cost-effectiveness analysis

Analyses were conducted in Stata v.13.0 [14]. Costs and outcomes were both discounted at 5% as per Australian health technology and decision-making bodies including the Pharmaceutical Benefits Advisory Committee and Medical Services Advisory Committee. The analysis was conducted from the perspective of the funder. A funder’s perspective has been adopted to reflect the underlying investment decision to provide the intervention or not. Whilst a societal perspective may illuminate the impact of the intervention on the broader health care system, it does not assist the decision maker (in this case the health insurer). Moreover, whilst the Australian health care landscape is a hybrid of private and public providers with both seeking to maximise the health of those they represent, they do so with differing constraints and substantially different optimisation problems. As such, a societal perspective is considered beyond the scope of this assessment. The time horizon of analysis was two years (from baseline to 24-month follow up).

#### Costs

Costs included in this analysis were the sum of benefits paid by the insurer and patient out of pocket costs associated with either services partially subsidised by the Government or for re-hospitalisation episodes. Regression analysis of the total (discounted) cost per patient as the dependent variable was conducted using a generalised linear model with trial group allocation (UC or HBI) as the predictive variable. A gamma family, log-link model was used given the skewed nature of health cost data. Age and sex were included as covariates. Costs are reported in Australian dollars and were adjusted to 2013 dollar terms using the total health price index[15].

The incremental cost per person randomised to receive the Young@Heart intervention was $1,196 charged to the private insurer. This did not include the cost of any subsequent consultations associated with any referrals initiated as part of the intervention.

#### Outcomes

Quality Adjusted Life Years (QALYs) were estimated based on completion of EuroQol—Five Dimension Three Level (EQ-5D-3L) Questionnaires[16] completed at baseline, 12 month follow-up and 24 month follow-up. Australian EQ-5D weights were applied to estimate utility values for each time point[17]. Those who died were allocated a utility value of 0 and missing data were excluded from analysis. Within trial QALYs were calculated using area under the curve analysis. Ordinary least squares regression analysis of individual QALYs was used to estimate the effect of receiving the intervention controlling for participants age and sex as covariates.

#### Within-trial Incremental Cost Effectiveness Ratio and Net Monetary Benefit

The incremental cost-effectiveness ratio (ICER) was estimated as the incremental costs divided by the incremental QALYs. In this analysis, this was achieved by dividing the beta weight associated with being allocated to the intervention group from the regression equation for cost by the beta weight associated with being allocated to the intervention from the regression equation for QALYs. The ICER was compared to a willingness to pay per QALY of $50,000 (Australian dollars), a commonly accepted value for health technology assessment in Australia [18]. Where the cost per QALY (i.e. the ICER) is below the threshold (here, $50,000), the intervention can be considered cost-effective, or represents ‘*acceptable value for money*’. However, where an ICER is negative or where the 95% confidence interval for either cost or effect includes 0, the preferred approach is to present the Incremental Net Monetary Benefit (INMB) instead of the ICER. The INMB is equal to the incremental QALYs multiplied by the willingness to pay per QALY less the incremental cost. The 95% confidence interval for the INMB was estimated using bootstrapping. In addition to assessing cost-effectiveness by age and sex, one-way sensitivity analyses were conducted with respect to the cost of the intervention ($600, $2,000 vs. base case estimate of $1,196).

### Modelled cost-effectiveness analysis beyond trial duration

**Fig 1:**
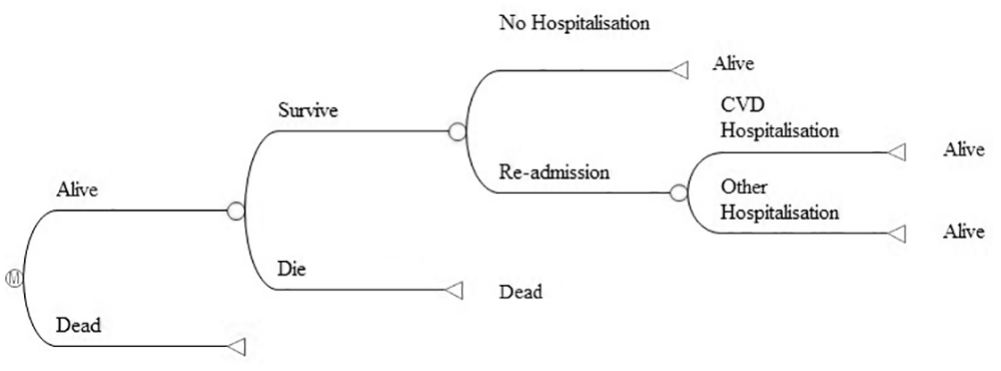
Overview of Model Structure. CVD: cardiovascular disease

A micro-simulation model (Fig 1) was constructed in TreeAge 2013 [19]. The model comprises of two health states, “Alive” and “Dead”. Simulated individuals begin in the “Alive” health state and face the probability of death, probability of re-hospitalisation and probability of remaining alive without having a re-hospitalisation. If an individual has a hospitalisation, the hospitalisation was either cardiovascular related or another cause. Risk of death, hospitalisation and hospitalisation type (i.e. transition probabilities) are dependent on simulated individuals’ age, sex, allocated treatment group (usual care or intervention) and count of previous re-hospitalisations (as they occur within the model). As the simulated individuals age or incur either a cardiovascular or other hospitalisation, their subsequent risk of death, re-hospitalisation and probability that a hospitalisation is cardiovascular related (i.e. transition probabilities) are revised. The advantage of a micro-simulation model of this nature enables transition probabilities, cost and health related quality of life utility values to be determined by the characteristics of the simulant (i.e. age, sex, treatment group) as well as the clinical history of the simulant (in this case number of hospitalisations and the reason for them). This allows a simplified model structure with only two health states (alive or dead) whereas an equivalent Markov model would require exponentially more. For example, a Markov model would require mutually exclusive health states for: each hospitalisation event (i.e. first re-hospitalisation, second re-hospitalisation); for both cardiac and other hospitalisation types (cardiac and other); as well as associated health-states to reflect every relevant combination of previous hospitalisations (i.e. post first cardiac hospitalisation, post second cardiac hospitalisation, post one cardiac hospitalisation and one non-cardiac hospitalisation and so forth). Simulated individuals were generated at random with respect to age and sex, based on respective distributions from baseline characteristics of the Young@Heart trial participants, and identical simulated individuals were passed through the model for both home based and usual care intervention.

Each model cycle was for one month to adequately reflect the timing of events observed in the trial. The duration of the simulation model was for 20 years, or quasi-lifetime given that the mean starting age of participants is 70. The analysis was conducted from the perspective of the funder. Internal validity of the simulation model was assessed by running the model for the same duration of the trial and comparing the simulated results with those of the trial.

#### Model parameters

**Transition Probabilities:** Transition probabilities were estimated using regression analysis of events (death, hospitalisation and hospitalisation type) at 1 month, months 2–6, months 7–12, months 12–24 and months 25 and over. This facilitated a non-linear risk of event over time. A Probit model was used for the dichotomous variable (event or no event) at each time point and for the distribution between cardiovascular or non-cardiovascular hospitalisation (given a hospitalisation). Hospitalisation events excluded dialysis and any other condition that the intervention was unlikely to affect (for example cancer) and were categorised as cardiovascular or non- cardiovascular related by expert nurse on review of the ICD-10, AR-DRG codes and case record notes. Hospitalisation episodes with the same admission date as the preceding hospitalisation discharge date were merged. The probability of an event and hospitalisation type was assessed with respect to participant age, sex, randomisation group and count of previous re-hospitalisations. Age, sex and treatment group transition probabilities were then predicted from the resulting regression equation along with the average marginal effect of a previous re-hospitalisation. Analyses were conducted in STATA 13.0 with robust standard errors. Participants who died in a prior analysis period were removed from estimation of subsequent risk of event calculations. Transition probabilities for beyond trial duration were extrapolated for the model based on the 25 month and over regression equations. There is considered no missing data for hospital or death events.

**Costs:** A gamma, log-link Generalised Estimating Equation model was used to account for the skew in episode cost data and multiple measures per trial participant. The costs included in this analysis were equivalent to those considered in the within trial analysis. For hospital episodes that were merged, the costs of each hospitalisation were totalled. The regression model estimated the cost per hospitalisation episode dependent on the age and sex of the trial participant, the count of previous re-hospitalisations and if the episode was cardiovascular related or not. The age-sex adjusted cost per episode of a cardiovascular and non-cardiovascular hospitalisation was estimated along with the incremental cost increase (or decrease) for each previous re-hospitalisation episode.

**Utility:** A Generalised Estimating Equation model was used to account for the multiple measures per trial participant. The regression model estimated utility dependent on age and sex of the trial participant, the count of previous re-hospitalisations for cardiovascular and the count of previous non-cardiovascular related re-hospitalisations. Based on the regression equation, age-sex specific utility scores were estimated along with the average marginal increase or decrease in utility associated with a cardiovascular or non-cardiovascular re-hospitalisation.

#### Modelled Incremental Cost Effectiveness Ratio

The incremental cost-effectiveness ratio (ICER) was estimated as the incremental costs divided by the incremental QALYs. In this analysis, QALYs are estimated as the time (number of cycles) multiplied by the simulated individuals’ utility score (at each cycle). Costs were accrued for each hospitalisation episode. The ICER was determined from the difference in mean costs and QALYs between simulated individuals receiving the home-based intervention or usual care. The simulation was undertaken for 1,000 simulated individuals.

**Sensitivity Analysis:** Distributions (standard errors) associated with each model parameter are provided from the regression equations. Probabilistic sensitivity analysis was conducted using Monte-Carlo simulation whereby the simulation model (of 1,000 individuals) is run 1,000 times. For each model run, a value for each model parameter was selected based on the corresponding distribution and provided an estimated ICER. A 95% confidence interval for the ICER was then estimated based on the 1,000 model runs.

## Results

### Within Trial Analysis Results

#### Costs

The predicted mean health care costs, controlling for age and sex, was $1,191 higher for the HBI group compared to UC ($28,839 compared to $27,648) (Table 1). However, HBI was associated with cost savings compared to UC for males aged over 75. This cost saving ($10,416) was greater than the cost to deliver the home based intervention ($1,196) (Table 1).

**Table 1:** Within Trial Predicted Mean Costs and QALYs: Home Based Intervention vs. Usual Care.

Sub-group	Home Based Intervention	Usual Care	Difference in predicted means	95% Confidence interval	
**Health care costs**					
**Male**					
≤64	$21,052	$21,658	-$606	-$9,881	$8,670
≥65 ≤74	$31,363	$26,995	$4,367	-$7,926	$16,660
≥75	$28,440	$38,598	-$10,158	-$26,474	$6,157
**Female**					
≤64	$23,552	$20,260	$3,292	-$15,256	$21,840
≥65 ≤74	$29, 131	$29,639	-$507	-$23,359	-$22,345
≥75	$40,125	$26,156	$13,969	-$4,953	$32,891
**Persons**	$28,803	$27,633	$1,171	-$5,042	$7,383
**Quality Adjusted Life Years**					
**Male**					
≤64	1.80	1.81	-0.01	-0.19	0.17
≥65 ≤74	1.48	1.64	-0.16	-0.33	0.01
≥75	1.42	1.50	-0.08	-0.27	0.12
**Female**					
≤64	1.67	1.75	-0.08	-0.43	0.27
≥65 ≤74	1.50	1.41	0.09	-0.23	0.41
≥75	1.44	1.46	-0.02	-0.25	0.21
**Persons**	1.56	1.62	-0.06	-0.15	0.03

Costs reported in Australian Dollars (2013)

QALY = quality adjusted life years

#### Outcomes

The predicted mean QALYs over the trial period, controlling for age and sex, was 0.06 lower for HBI compared to UC (1.55 compared to 1.62) (Table 1). However, HBI was associated with higher QALYs compared to UC for females aged between 65 and 75. However, none of the differences in QALYs was statistically significantly different.

#### Within-trial Incremental Cost Effectiveness Ratio and Net Monetary Benefit

The ICER for HBI compared to UC is negative (Table 2). Given the difficulties interpreting negative ICERs, the net monetary benefit was also estimated. Based on a willingness to pay threshold of $50,000 per QALY, HBI was associated with a negative net monetary benefit. However, for males aged over 75 and females aged between 65 and 75, HBI was associated with positive INMB. Although for both sub-groups, the estimated net monetary benefit was associated with a significant degree of uncertainty. The results of the within trial analysis are not significantly altered when the analysis is conducted with alternative intervention costs.

**Table 2:** Within Trial ICER and INMB, Home Based Intervention vs. Usual Care.

Population	ICER	INMB		
		Mean	(95% CI)	
**Male**				
≤64	$53,589^2^	$41	-$15,600	$15,681
≥65 ≤74	-$27,551^1^	-$12,293	-$27,310	$2,724
≥75	$133,428^2^	$6,352	-$10,965	$23,668
**Female**				
≤64	-$42,021^1^	-$7,209	-$38,082	$23,663
≥65 ≤74	-$5,660^3^	$4,990	-$23,081	$33,061
≥75	-$725,691^1^	-$14,931	-$35,473	$5,610
**Persons**	-$19,001^1^	-$4,251	-$12,025	$3,524
**Sensitivity Analysis**				
**Cost of intervention:**				
$600	-$9,326^1^	-$3,655	-$11,429	$4,120
$2,000	-$32,052^1^	-$5,055	-$12,829	$2,720

CI = confidence interval; ICER = incremental cost effectiveness ratio; INMB = incremental net monetary benefit

^1^ More costly and reduced Quality Adjusted Life Years

^2^ Less costly but reduced Quality Adjusted Life Years

^3^ Less costly and increased Quality Adjusted Life Years

### Simulation Model Results

#### Model parameters

Model parameters were derived using the predicted means based on regression analyses of trial data. Results of the regression analyses are provided in Supporting Information (S1 File).

**Transition probabilities:** The intervention was not a significant predictor of hospitalisation or death. HBI was associated with less probability of a hospitalisation being cardiovascular; however this was only statically significant for hospitalisations in the first month and for months 7 to 18 (Supporting Information S1 File). The number of previous cardiovascular or other hospital admissions statistically significantly increased the probability of a re-hospitalisation from months 24 and onwards but not for earlier time points. The number of previous cardiovascular admissions were associated with a significant increase in the probability of subsequent hospitalisations being cardiovascular related. Previous hospitalisations for other causes were associated with a statistically significant decrease in subsequent hospitalisations being cardiovascular. There was no statistically significant relationship between the number of previous hospitalisation episodes (cardiovascular or other) and the probability of death.

**Costs:** Patient age and type of hospitalisation (cardiovascular) were statistically significantly associated with a higher per episode cost of hospitalisation (Table 3). In addition, the number of previous non-cardiovascular related hospitalisations were associated with a statistically significant reduction in the per episode cost of subsequent hospitalisations. For probabilistic sensitivity analysis, the cost of a hospitalisation and incremental cost per previous hospitalisation were sampled from a gamma distribution.

**Table 3:** Predicted Mean Cost per Hospitalisation and Incremental Cost Associated with Number of Previous Hospitalisations.

Age	Cost of CV hospitalisation, Mean, (SE)	Increased cost of CV hospitalisation per previous hospitalisation, Mean, (SE)	Cost of Other hospitalisation, Mean, (SE)	Increased cost of other hospitalisation per previous hospitalisation, Mean, (SE)
**Female**				
40	$8,796 ($2,108)	$340 ($147)	$6,051 ($1,238)	$234 ($94)
50	$10,006 ($2,035)	$387 ($159)	$6,883 ($1,111)	$266 ($101)
60	$11,382 ($2,002)	$440 ($174)	$7,830 ($985)	$303 ($110)
70	$12,947 ($2,091)	$500 ($194)	$8,907 ($944)	$344 ($122)
80	$14,727 ($2,412)	$569 ($220)	$10,131 ($1,120)	$391 ($139)
90	$16,752 ($3,050)	$647 ($254)	$11,525 ($1,580)	$445 ($161)
**Male**				
40	$10,872 ($1,963)	$420 ($168)	$5,142 ($866)	$199 ($76)
50	$12,367 ($1,729)	$478 ($183)	$5,849 ($716	$226 ($82)
60	$14,067 ($1,546)	$544($201)	$6,653 ($565)	$257 ($90)
70	$16,002 ($1,617)	$618 ($226)	$7,568 ($536)	$292 ($100)
80	$18,202 ($2,149)	$703 ($259)	$8,609 ($790)	$333 ($115)
90	$20,705 ($3,157)	$800 ($304)	$9,793 ($1,292)	$378 ($135)

Costs reported in Australian Dollars (2013)

CV = cardiovascular; Std. Error = robust standard error

**Utilities:** Patient age and number of hospitalisations were associated with a statistically significantly lower health related quality of life score (Table 4). Cardiovascular hospitalisations were associated with a 0.022 mean reduction in health related quality of life compared to a 0.012 reduction for other hospitalisations. For probabilistic sensitivity analysis, utility values were sampled from a beta distribution whereas disutility associated with a hospitalisation event were sampled from a gamma distribution.

**Table 4:** Predicted Health Related Quality of Life Utility and Disutility Estimates.

	Predicted Mean	Std. Error	Distribution
**Age**			
40	0.97	0.02	Beta
50	0.93	0.02	Beta
60	0.89	0.01	Beta
70	0.85	0.01	Beta
80	0.81	0.01	Beta
90	0.77	0.02	Beta
**Event**			
CV hospitalisation	-0.022	0.010	Gamma
Other hospitalisation	-0.012	0.003	Gamma

CV = cardiovascular; Std. Error = robust standard error

#### Modelled Incremental Cost Effectiveness Ratio

The model predicts overall survival and number of hospitalisations (by type) for the two treatment groups based on the input parameters described above. HBI was associated with more cardiovascular hospitalisations and fewer other hospitalisations (Table 5). Overall, HBI was associated with an increased cost ($9,288) and fewer QALYs (-0.10) than usual care, thus resulting in a negative ICER of $97,506.

**Table 5:** Model Outcomes and Incremental Cost-Effectiveness Ratio Over 20 Years.

Outcome	Usual Care	Home Based Intervention	Home based Intervention vs. Usual Care
Death, n (%)	228	233	5
Hospitalisations per person			
CV	1.67	2.63	0.96
Other	3.59	3.41	(0.18)
Cost per person, mean (95% CI)	$34,759	$44,046	$9,288 ($2,884, $16,252)
QALYs per person, mean (SD)	9.11	9.02	(0.10) (-0.79, 0.56)
Incremental Cost Effectiveness Ratio			($97,506)
Net Monetary Benefit			($10,172)

**Fig 2:**
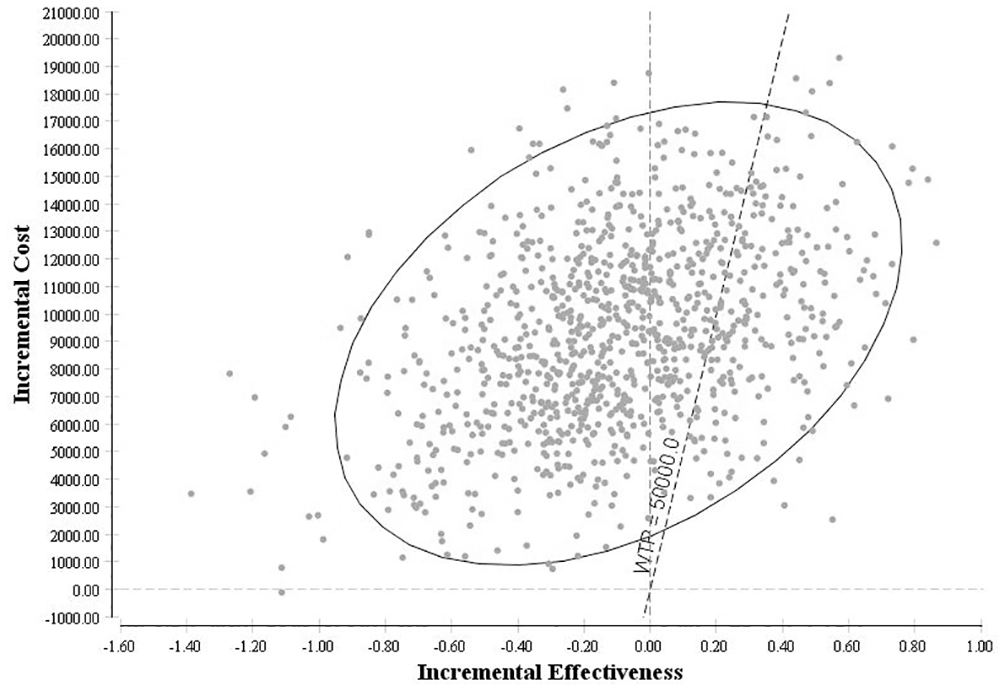
Probabilistic Sensitivity Analysis for Cost-effectiveness of Home Based Intervention vs. Usual Care. Each dot represents a modelled incremental cost-effectiveness ratio; the eclipse represents the 95% credible interval; the dashed line represents cost-effectiveness ratio equal to $50,000 per quality adjusted life year. HBI: home based intervention. WTP: willingness to pay threshold. Incremental Effectiveness measured in Quality Adjusted Life Years, Incremental Cost measured in Australian Dollars (2013)

CI = confidence interval; CVD = cardiovascular; SD = standard deviation;

The greater majority of model iterations (61.1%) resulted in HBI being determined inferior to UC. Of the remaining model iterations, 20.5% resulted in an ICER of less than $50,000 and 18.3% resulted in an ICER greater than $50,000 (Fig 2).

For males, the modelled results indicate an overall cost saving associated with the HBI (after accounting for the cost of the intervention), however it was also associated with fewer QALYs (Table 6). For females, the modelled results estimated HBI being associated with higher costs and fewer QALYs.

**Table 6:** Modelled ICER and INMB, Home Based Intervention vs. Usual Care.

Population	Δ Cost	Δ QALY	ICER	INMB		
				Mean	Lower 95% CI	Upper 95% CI
**Male**						
55	-$1,155	-0.09	$12,291^2^	-$3,542	-$136,168	-$2,793
65	-$1,664	-0.13	$12,424^2^	-$5,032	-$154,094	-$4,812
75	-$1,980	-0.15	$12,997^2^	-$5,636	-$80,693	-$3,948
**Female**						
55	$6,104	-0.09	-$70,901^1^	-$10,407	-$13,951	-$4,570
65	$6,625	-0.07	-$95,218^1^	-$10,104	-$8,401	-$4,837
75	$7,023	-0.08	-$89,172^1^	-$10,960	-$6,283	-$1,725

CI = confidence interval; ICER = incremental cost effectiveness ratio; INMB = incremental net monetary benefit; QALY = quality adjusted life years; Δ = incremental

^1^ More costly and reduced Quality Adjusted Life Years

^2^ Less costly but reduced Quality Adjusted Life Years

## Discussion

Based on the overall results of the Young@Heart Study, the application of a home-based secondary prevention program to supplement high quality usual care for privately insured patients with chronic heart disease in Australia was associated with no statistically significant or clinically meaningful changes in quality adjusted life years or in health care costs. Both the within trial and modelled cost-effectiveness analyses conducted in this study indicate that the HBI did not provide a statistically significant increase in quality adjusted life years. However, consistent with the original report of gender differentials in outcome, for males (representing around two thirds of the cohort) and in particular males 75 years and over (42% of males) HBI indicated a potential to reduce health care costs when compared to usual care (within trial: -$4,662 [95%CI: -$18,324, $8,999]; modelled analysis: -$1,980 [95%CI: -$22,843, $14,863]). Thus, a clear need to identify the population that will benefit the most from a management program has emerged.

However, these results are not in keeping with previous estimates of the cost-effectiveness of disease management programs, but may reflect the specific population studied (i.e. those privately insured). A recent review [18] of model based analyses reporting the cost-effectiveness of cardiovascular disease management programs found all included studies (n = 16) provided improved health benefits relative to usual care with six of these studies also identifying disease management programs as cost-saving. This may be explained by potentially diminishing marginal returns associated with new disease management programs. That is, as disease management programs, or aspects of them, become increasingly implemented into usual care, the marginal benefits of improved programmes may be less pronounced. Alternatively, as postulated in the primary report, it may well reflect the health care environment in which the intervention was applied and/or differences in the level of engagement with the cardiac nurses, (this is the subject of ongoing analyses). Confirming the primary analyses, these data demonstrated that differential patient cohorts (based on age and sex) may achieve relatively different outcomes from the implementation of a disease management program. In the face of diminishing marginal returns, the identification of patients most likely to benefit from disease management programs will become increasingly important to ensure that the implementation of such programs are cost-effective. Notably, the disease management program appeared to be most cost-effective in older men and this demographic cohort represents the most predominant patient group presenting with chronic heart disease.

This program has been investigated within an Australian population with private health insurance. There remains significant points of differentiation between the Australian private and public health care sectors[20] including patient socioeconomic and demographic characteristics (including better self-reported health[21]). As such, the results from this analysis may not necessarily be transferable to the broader public health care system; particularly given favourable results from similar disease management programs targeting those with chronic heart disease[22] and atrial fibrillation[23] in Australia. Moreover, the incremental benefit associated with disease management programmes may, in Australia at least, be greater in the public health care system than for those with private insurance; indeed a similar program implemented in an equivalent cohort of cardiac patients in that setting demonstrated markedly reduced hospital stay in both men and women [24].

This study is subject to a number of limitations that should be acknowledged. Firstly, changes in pharmaceutical and medical service costs were not captured in this analysis. Whilst it is likely that a home based intervention may provide the necessary support and tools to optimise pharmacological treatment, the cost implications are unclear. On the other hand, disease management programs could be associated with increased costs from higher utilisation of medical services due to: increased level health screening, awareness and where providing referrals to supplementary services are elements of the intervention itself. As such, these estimates may underestimate the total costs associated with the home-based intervention in this study. Secondly, this analysis has focused on the health related quality of life information collected during the trial and modelled future health related quality of life with respect to hospitalisations. This approach, whilst best representing the clinical trial evidence, does not include other significant improvements associated with the home based intervention from the clinical trial (including reductions in: body weight, waist circumference, total cholesterol, systolic and diastolic blood pressure). However, none of these improvements are significantly different to those achieved with usual care. Thus, whilst the fullness of benefits may not have been captured in this analysis, the incremental benefits of home based intervention compared to usual care is not likely to be significantly biased. Finally, the model considered hospitalisation and death as mutually exclusive events. For those that die during a hospitalisation event, the model considers these to have occurred in two separate cycles. Given that the cycle length within the model is 30 days it is unlikely that this will significantly alter the results estimated. Moreover, there is no reason to believe that such cases occurred more or less frequently in the intervention arm compared to usual care and as such, will not bias the incremental results presented here.

Despite these limitations, this work provides a crucial impetus for the continuation of efforts to improve the evidence base regarding appropriate patient selection and design of disease management programs. Future innovation within disease management programs is likely to be driven by *a priori* identification and targeting of disease management programs, or their components, to patients most likely to achieve benefits that outweigh the costs of the program; with particular consideration of the health care system within which the program is being applied.

## Supporting Information

S1 File Parameters for microsimulation model including: transition probabilities, costs and health related utility estimates. (PDF)
