# Supplementary material for: Cost-Effectiveness of a Home Based Intervention for Secondary Prevention of Readmission with Chronic Heart Disease
Source: PLoS One. 2015 Dec 10;10(12):e0144545. doi: 10.1371/journal.pone.0144545 (PMC4684189; doi:10.1371/journal.pone.0144545)
Supplement: S1 File — (PDF) [file pone.0144545.s001.pdf]

Supporting Information

Transition probabilities

Probability of hospitalisation

Table S1 Transition probabilities for hospitalisation dependent on Intervention group, Age, Sex and previous hospitalisations

Month	Group (Std. Error)	Age (Std. Error)	Sex (Std. Error)	Previous CV hospital (Std. Error)	Previous Other hospital (Std. Error)
1	- 0.05 (0.10)	-0.01 (0.01)*	0.01 (0.11)	N/A	N/A
2-6	-0.14 (0.12)	0.001 (0.005)*	0.08 (0.10)	-0.21 (0.07)*	-0.10 (0.06)
7-12	-0.06 (0.09)	0.006 (0.004)	-0.01 (0.09)	-0.05 (0.03)	-0.02 (0.02)
13-18	0.08 (0.08)	-0.002 (0.004)	0.03 (0.09)	-0.04 (0.03)	-0.03 (0.01)
19-24	0.06 (0.06)	0.009 (0.004)*	-0.02 (0.08)	0.02 (0.02)	-0.00 (0.00)
25+	0.11 (0.10)	-0.001 (0.005)	0.02 (0.08)	0.09 (0.02)*	0.02 (0.01)*

CV = cardiovascular; N/A = not applicable; Std. Error = Robust Standard Error; * indicates statistical significance at the 0.05 level

Probability hospitalisation is CVD related

Table S2 Probability hospitalisation is CVD related dependent on Intervention group, Age, Sex and previous hospitalisations

Month	Group (Std. Error)	Age (Std. Error)	Sex (Std. Error)	Previous CV hospital (Std. Error)	Previous Other hospital (Std. Error)
1	-0.55 (0.25)*	-0.02 (0.02)	0.14 (0.28)	N/A	N/A
2-6	0.05 (0.15)	0.002 (0.008)	0.05 (0.17)	0.87 (0.23)*	-1.81 (0.41)*
7-12	-0.33 (0.16)*	-0.003 (0.008)	0.28 (0.19)	0.46 (0.11)*	-0.24 (0.09)*
13-18	-0.34 (0.15)*	0.012 (0.008)	-0.15 (0.17)	0.33 (0.06)*	-0.29 (0.08)*
19-24	-0.13 (0.17)	0.16 (0.010)	-0.22 (0.18)	0.23 (0.04)*	-0.17 (0.07)*
25+	-0.26 (0.14)	0.002 (0.007)	-0.18 (0.14)	0.22 (0.06)*	-0.05 (0.01)*

CV = cardiovascular; N/A = not applicable; Std. Error = Robust Standard Error; * indicates statistical significance at the 0.05 level

Probability of Death

Table S3 Probability of death dependent on Intervention group, Age, Sex and previous hospitalisations

Month	Group (Std. Error)	Age (Std. Error)	Sex (Std. Error)	CV or Other hospital (Std. Error)
1	-0.51 (0.46)	0.08 (0.03)*	0.38 (0.48)	N/A
2-6	0.85 (0.47)	0.07 (0.03)*	0.74 (0.56)	-0.25 (0.13)
7-12	0.58 (0.39)	0.02 (0.02)	0.04 (0.36)	0.007 (-0.01)
13-18	0.26 (0.26)	0.02 (0.01)	0.63 (0.39)	0.001 (0.01)
19-24	0.02 (0.34)	0.06 (0.02)*	0.19 (0.38)	-0.06 (0.06)
25+	0.13 (0.30)	0.02 (0.02)	0.49 (0.44)	0.01 (0.01)

CV = cardiovascular; N/A = not applicable; Std. Error = Robust Standard Error; * indicates statistical significance at the 0.05 level

Costs

Table S4 Cost per hospitalisation dependent on Age, Sex, reason for hospitalisation and previous hospitalisations

Constant (Std. Error)	Age (Std. Error)	Sex (Std. Error)	CV Related (Std. Error)	Previous Other Hospital (Std. Error)	CV Related # Male Interaction (Std. Error)
12.80 (0.40)*	0.013 (0.005)*	-0.16 (0.12)	0.39 (0.18)*	0.04 (.01)*	0.36 (0.22)

CV = cardiovascular; Std. Error = Robust Standard Error; * indicates statistical significance at the 0.05 level

Utilities

Table S5 Predicted Health Related Utility Values

Variable	Coefficient	Std. Err	95% Confidence Interval	
Age	-0.004 *	0.001	-0.005	-0.003
Male	0.040 *	0.014	0.013	0.067
CV re-hospitalisations	-0.067 *	0.018	-0.102	-0.033
Other rehospitalisation	-0.022 *	0.006	-0.034	-0.010
Time	0.000	0.000	-0.000	0.001
Time # CV re-hospitalisation	0.002 *	0.001	0.001	0.003
Time # Other re-hospitalisation	0.001 *	0.000	0.000	0.001
Constant	1.120 *	0.048	1.026	1.215

CV = cardiovascular; Std. Error = Robust Standard Error; * indicates statistical significance at the 0.05 level
